# Melatonin as an immunomodulator in CD19-targeting CAR-T cell therapy: managing cytokine release syndrome

**DOI:** 10.1186/s12967-023-04779-z

**Published:** 2024-01-14

**Authors:** Na Zheng, Yihao Long, Zixuan Bai, Jianing Li, Hongyu Wang, Dan-Dan Song, Hong-Lin Liu, Jian-Hong Shi, Shuli Zhao

**Affiliations:** 1 General Clinical Research Center, Nanjing First Hospital, Nanjing Medical University, Nanjing, Jiangsu China; 2 Department of Cell Biology, School of Basic Medical Sciences, Nanjing Medical University, Nanjing, Jiangsu China; 3 Central Laboratory, Hebei Collaborative Innovation Center of Tumor Microecological Metabolism Regulation, Hebei Key Laboratory of Cancer Radiotherapy and Chemotherapy, Affiliated Hospital of Hebei University, Baoding, Hebei China; 4 Institute of Clinical Medical Sciences, China–Japan Friendship Hospital, Beijing, China

**Keywords:** Melatonin, Chimeric antigen receptors, Cytokine release syndrome, Cell- and tissue-based therapy, CD19 antigen, Adoptive immunotherapy, T lymphocytes

## Abstract

**Background:**

Chimeric antigen receptor CAR-T cell therapies have ushered in a new era of treatment for specific blood cancers, offering unparalleled efficacy in cases of treatment resistance or relapse. However, the emergence of cytokine release syndrome (CRS) as a side effect poses a challenge to the widespread application of CAR-T cell therapies. Melatonin, a natural hormone produced by the pineal gland known for its antioxidant and anti-inflammatory properties, has been explored for its potential immunomodulatory effects. Despite this, its specific role in mitigating CAR-T cell-induced CRS remains poorly understood.

**Methods:**

In this study, our aim was to investigate the potential of melatonin as an immunomodulatory agent in the context of CD19-targeting CAR-T cell therapy and its impact on associated side effects. Using a mouse model, we evaluated the effects of melatonin on CAR-T cell-induced CRS and overall survival. Additionally, we assessed whether melatonin administration had any detrimental effects on the antitumor efficacy and persistence of CD19 CAR-T cells.

**Results:**

Our findings demonstrate that melatonin effectively mitigated the severity of CAR-T cell-induced CRS in the mouse model, leading to improved overall survival outcomes. Remarkably, melatonin administration did not compromise the antitumor effectiveness or persistence of CD19 CAR-T cells, indicating its compatibility with therapeutic goals. These results suggest melatonin's potential as an immunomodulatory compound to alleviate CRS without compromising the therapeutic benefits of CAR-T cell therapy.

**Conclusion:**

The study's outcomes shed light on melatonin's promise as a valuable addition to the existing treatment protocols for CAR-T cell therapies. By attenuating CAR-T cell-induced CRS while preserving the therapeutic impact of CAR-T cells, melatonin offers a potential strategy for optimizing and refining the safety and efficacy profile of CAR-T cell therapy. This research contributes to the evolving understanding of how to harness immunomodulatory agents to enhance the clinical application of innovative cancer treatments.

## Introduction

With its extraordinary potency and sustained clinical response, chimeric antigen receptor (CAR-T) cell therapy represents a breakthrough in the treatment of hematologic cancer. CARs, which are engineered synthetic receptors, enable the redirection of lymphocytes, primarily T cells, to recognize and destroy cells expressing specific target antigens [1]. The US Food and Drug Administration (FDA) approved anti-CD19 CAR-T cell therapy for B-cell malignancies in 2017 due to its outstanding effectiveness [2]. Furthermore, the therapeutic potential of CAR T-cell therapy is also being expanded by the novel CAR designs that now undergo clinical testing and target alternative cancer antigens.

CAR-T cell therapy exhibits complex in vivo pharmacokinetics influenced by intrinsic and extrinsic factors such as product phenotype, composition, tumor burden, and prior lymphodepletion treatments. Moreover, the functional activity of CAR-T cells differs between patients, making it challenging to establish dosing regimens with consistent efficacy and manageable toxicity [3]. Consequently, the clinical application of CAR-T cell therapy is currently restricted to medically fit patients at specialized cancer centers due to associated acute and chronic side effects. The most prevalent acute toxicity observed in CAR-T cell therapy is cytokine release syndrome (CRS), which is triggered by the release of inflammatory cytokines from CAR-T cells, leading to the production of critical cytokines like interleukin 6 (IL-6) by innate immune cells [4]. Current management strategies for CRS include IL-6 neutralization through receptor antagonists and systemic immunosuppression using steroids [5]. However, the efficacy and safety of these approaches are limited by their high cost [6] and potential impairment of the infused CAR-T cells' anti-tumor efficacy [7].

Given these challenges, novel adjunctive therapeutic strategies that are safe, affordable, and easily implemented are urgently needed to improve CAR T-cell therapy clinical outcomes. Melatonin, a naturally occurring hormone primarily synthesized by the pineal gland, possesses anti-inflammatory and immunomodulatory properties [8]. Notably, melatonin has been found to inhibit the production and release of pro-inflammatory cytokines, including interleukin-6 (IL-6) and interleukin-1β (IL-1β). Some of these actions are certainly mediated by melatonin membrane receptors, such as MT1 and MT2. Melatonin effectively scavenges a wide range of reactive oxygen/nitrogen species (ROS/RNS), including hydroxyl radicals and the commonly overlooked carbonate radical [9]. Melatonin administration inhibits the secretion of proinflammatory factors in a murine model of atherosclerosis and subarachnoid hemorrhage [10]. Similarly, melatonin reduces the serum concentration of pro-inflammatory cytokines and oxidative stress markers in patients with relapsing–remitting multiple sclerosis (RRMS) [11]. Consequently, melatonin has emerged as a potential therapeutic agent for managing cytokine storms, including those associated with CAR T-cell therapies. Furthermore, melatonin exhibits its anticancer effects throughout various stages of tumor development, including initiation, promotion, and progression, while preserving the integrity of normal cells [12–15]. Although the precise mechanisms underlying the pro-apoptotic selectivity and efficiency of melatonin in cancer cells are not fully understood, melatonin likely modulates unique biochemical pathways associated with cancer [16]. These findings underscore the remarkable potential of melatonin as a promising candidate for selectively targeting cancer cells while preserving the normal cells and tissues, aligning with a long-envisioned strategy for managing side effects.

Extensive preclinical studies have greatly facilitated the development and optimization of anti-CD19 CAR-T cells, while cell line-derived xenograft models have been vital for evaluating therapy efficacy and safety. Among the numerous available models, mice xenografted with Raji tumor cells have gained widespread recognition and acceptance for testing CD19-targeting CAR-T cell therapies [17]. Consequently, this study aimed to use Raji tumor cells and mouse models to investigate the role of melatonin as an immunomodulatory compound in CD19-targeting CAR T-cell therapy, thereby providing valuable insights into improving treatment protocols and ultimately enhancing patient outcomes.

## Materials and methods

### Antibodies and reagents

Fluorescence-labeled antibodies for CD3(SK7), CD4 (RM4-5), CD8 (53–6.7), CD11b (M1/70), CD25 (PC61.5), CD44 (IM7), CD62L (MEL-14), CD45 (30-F11), CD69 (H1.2F3), iNOS (CXNFT), and F4/80 (J43) were purchased from eBioscience. FITC-conjugated F(ab')2 fragment of goat anti-human IgG1 Fcγ antibody (Jackson ImmunoResearch Laboratories, West Grove, PA) was utilized to detect anti-CD19 CAR positive cells. Anti-CD3/anti-CD28 (αCD3/αCD28) monoclonal antibodies were purchased from eBioscience. Melatonin was purchased from Sigma (M5250).

### Cell culture

Burkitt’s Lymphoma Raji cells were obtained from the American Tissue Culture Collection (ATCC). To establish single-cell clones expressing luciferase, wild-type tumor cell lines were genetically modified through stable transduction with a lentiviral vector containing the firefly luciferase (FLuc) gene encoding firefly luciferase (Lentigen Technology). Following transduction, the luciferase-expressing cells were selectively isolated. Raji-FLuc cells were cultured in RPMI medium (Invitrogen) supplemented with 10% FBS (Sigma-Aldrich) and, 2 mM GlutaMAX (Thermo Fisher Scientific).

### Cell viability assay

Cell viability was determined using the 3-(4,5-dimethylthiazol-2-yl)-2,5-diphenyltetrazolium bromide (MTT) assay as previously described [18]. Briefly, Raji cells in the exponential growth phase were seeded into 96-well plates. The cells were then exposed to melatonin at concentrations ranging from 0 to 10 mM for 24 h, as well as at different time points (0–24 h) with a fixed concentration of 10 mM. The absorbance of formazan solution was measured at 570 nm after each treatment period using a microplate reader (Multiskan FC, Thermo Scientific).

### T cell isolation and activation

Mouse T cells were isolated from the spleen and lymph nodes using CD90.2 MicroBeads (Miltenyi Biotec). Enriched T cells were subjected to flow cytometric cell sorting, targeting the CD3 + CD44loCD62Lhi surface markers, to isolate purified naïve T cells. These purified naïve T cells were cultured in RPMI-1640 medium supplemented with 10% FBS, 2 mM GlutaMAX, and subsequently activated for T cell activation analysis using plate-bound anti-CD3 (1 μg/ml) and anti-CD28 (1 μg/ml) antibodies. In the in vitro experiment, melatonin (1 mM) was administered simultaneously with T cell receptor (TCR) stimulation of T cells.

Buffy coats from anonymous healthy donors were obtained from the Blood Center of Jiangsu Province. All necessary ethical and safety protocols were followed during the handling of buffy coats. A positive selection method was used to isolate CD4-positive and CD8-positive human T cells. This process involved using a 1:1 mixture of CD4-negative and CD8-negative microbeads (Miltenyi Biotec) following the manufacturer's protocol. Isolated T cells were plated onto 24-well tissue culture plates (Corning) precoated with 1 μg/mL anti-CD3 antibody and 1 μg/mL anti-CD28 antibody in RMPI medium supplemented with 10% FBS, 20 IU IL-2 (130–097-745, Miltenyi) and 10 ng/mL IL-7 (130–095-363, Miltenyi), 2 mM GlutaMax.

### Mice

The mice were treated in accordance with all relevant animal use guidelines and ethical regulations based on a protocol approved by the Animal Care Committee of Nanjing First Hospital, Nanjing Medical University. In the CRS model, 6–8-week-old female CB17.Cg-PrkdcscidLystbg-J/CrlBltw (SCID ® beige) mice were intraperitoneally injected with 3 × 10^6^ Raji-Fluc cells, and tumors were allowed to grow for 20 days. Melatonin or phosphate-buffered saline (PBS, vehicle) was administered intraperitoneally at 10 mg/kg once per day, beginning 5 h before CAR T cell transfer. For the next three days, inject once every 24 h, a total of four injections. To assess the function of CD19-targeting CAR-T cells in vivo, we utilized 6–8-week-old NSG mice. Mice were injected intravenously (i.v.) with 6 × 10^6^ Raji luciferase cells on day 0. Same as the CRS model, Melatonin or phosphate-buffered saline (PBS, vehicle) was administered intraperitoneally at 10 mg/kg once per day, beginning 5 h before CAR T cell transfer. For the next 3 days, inject once every 24 h, a total of four injections. The choice of administering melatonin at a dose of 10 mg/kg in the drinking water to the aged mice was based on previous literature and research findings that suggest this dosage may have beneficial effects in reducing inflammation [19, 20]. Tumor burden in mice was assessed by intraperitoneal injection of Raji-Luc cells followed by imaging using the IVIS Spectrum bioluminescence system (PerkinElmer, USA). Before imaging, mice received an intraperitoneal injection of 10 μl/g body weight of 15 mg/ml D-luciferin potassium salt (Beyotime, Shanghai, China) dissolved in PBS.

### Virus production and transduction of T cell

The virus was generated through Lipofectamine-mediated transient transfection of 293 T cells with plasmids containing Moloney murine leukemia virus gag-pol, RD114 envelope, and transfer vector. For transduction of human T cells, isolated T cells were plated onto 24-well tissue culture plates precoated with 1 μg/mL anti-CD3 antibody and 1 μg/mL anti-CD28 antibody in RMPI medium supplemented with 10% FBS, 20 IU IL-2 and 10 ng/mL IL-7, 2 mM GlutaMax on day 0. After 2 days, T cells were transduced with retroviral supernatants through centrifugation on Retronectin-coated plates, resulting in the generation of CD19-CAR T cells. Transduced T cells were cultured at a concentration of 0.5 × 10^6^ cells/ml in T cell medium enriched from day 2 after transduction onwards. T cells were typically counted every 2 days. T cell expansion was calculated by dividing the absolute number of expanded T cells at each time point during culture by the respective number on day 0 (T cell transduction). T cell viability was assessed by trypan blue staining. Transduction efficiency was confirmed three days later via flow cytometry (gated on CD3 + T cells). CAR-T cells were administered to mice seven days following the initial T-cell activation. Culture medium was changed and cell factor replenished every 1 to 2 days.

### Serum collection

Blood samples were obtained from the mice using either the tail clip or the retro-orbital bleeding method. The collected blood was allowed to clot for 30 min at 37 °C. To separate serum, the clotted blood samples were centrifuged at 6000 ×*g* for 10 min at 4 °C. For optimal storage conditions, the resulting serum was divided into aliquots and placed into individual tubes to prevent multiple freeze–thaw cycles. Finally, the aliquoted serum samples were promptly stored at a temperature of − 80 °C until further analysis to ensure sample integrity.

### Cytokine measurements

Serum cytokines were measured using ELISA kits for mouse SAA3 (Millipore), IL-6, IL-1β, and human IL-2, IL-10, IFN-γ (Thermo Fisher Scientific) according to the manufacturer’s instructions.

During the cytokine measurement assay, T cells were prepared by replacing the culture medium with cytokine-free medium 24 h in advance. This step ensured that the T cells were devoid of any residual cytokines or interference from the culture medium, allowing for accurate cytokine measurements.

### Isolation of murine peritoneal macrophages

Peritoneal macrophages were isolated following a standard procedure [21]. Briefly, mice were euthanized by CO2 inhalation, and approximately 5 mL of ice-cold PBS was gently injected into the peritoneal cavity using a 25-gauge needle. Abdominal massaging was performed to aid the dispersion of peritoneal cells. The peritoneal fluid was then aspirated utilizing an 18-gauge needle and carefully collected into a sterile tube. The collected peritoneal fluid underwent centrifugation at 300 ×*g* for 5 min at 4 °C to pellet the cells. The resulting cell pellet was resuspended in complete RPMI-1640 medium, which included 10% FBS, 1% penicillin–streptomycin, and 2 mM GlutaMax. Subsequently, the isolated peritoneal macrophages were counted using a hemocytometer, and cell viability was assessed through trypan blue exclusion. The identity of peritoneal macrophages was confirmed by flow cytometry, characterized by high expression of F4/80 and CD11b. Further analysis included gating for iNOS expression.

### Flow cytometry and intracellular cytokine staining (ICS)

Flow cytometric analyses were performed using a DxFLEX flow cytometer (Beckman) as described previously [22]. For intracellular cytokine staining (ICS), the cells were first stimulated with 50 ng/ml PMA plus 750 ng/ml ionomycin for 6 h in the presence of 10 μg/ml protein transport inhibitor monensin (eBioscience). Subsequently, the cells were fixed and incubated with the appropriate antibodies as indicated. Finally, flow cytometry was employed to analyze the stained cells.

### Statistical analyses

Statistical analyses were performed using GraphPad Prism software. Significant differences between the two groups were analyzed using unpaired two-tailed Student’s t-tests. Kaplan–Meier analyses were performed, and the log-rank Mantel-Cox test was used to determine the statistical differences between the survival curves for the two groups. P values < 0.05 were considered significant, and the levels of significance were indicated as *P < 0.05, **P < 0.01, and ***P < 0.001. All data are presented as the mean ± SEM.

## Results

### Concentration-dependent effects of melatonin on Raji cell line and primary T cells

**Fig. 1:**
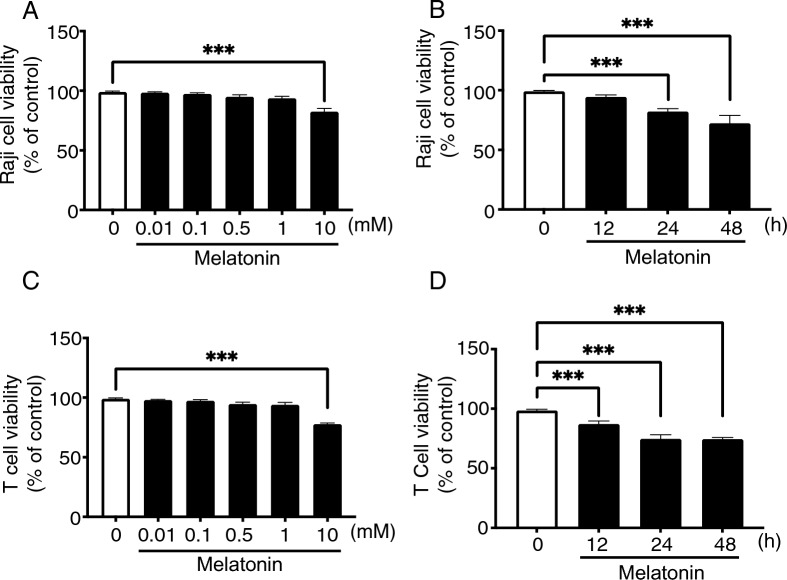
Melatonin effects on the Raji cell line and primary T cells. **A** Raji cells were subjected to treatment with melatonin at varying concentrations ranging from 0 to 10 mM for a duration of 24 h. **B** Raji cells were incubated with 10 mM melatonin for various time-points. The cell viability was measured by MTT assay. **C** T cells were subjected to treatment with melatonin at varying concentrations ranging from 0 to 10 mM for a duration of 24 h in the presence of interleukin-2 (IL-2, 100 IU/ml). **D** T cells were incubated with 10 mM melatonin for various time-points in the presence of IL-2 (100 IU/ml). The cell viability was measured by MTT assay. Data are representative of 3 independent experiments. Summary graphs (N = 4) are presented as mean ± SEM, P values were determined by unpaired two-tailed Student’s *t*-test. *P < 0.05; **P < 0.01; ***P < 0.001

The effects of melatonin on the Raji cell line and primary T cells were investigated by exposing the cells to various concentrations of melatonin and assessing their responses. The concentration-dependent effects of melatonin on cell viability and the induction of cell death were examined. Remarkably, no significant changes in Raji cells or primary T cells viability were observed after 24 h of treatment with melatonin concentrations ranging from 0.01 nM to 1 mM (Fig. 1A and C). However, high melatonin concentrations (10 mM) elicited a distinct response, leading to cell death (Fig. 1A and C). Furthermore, the extent of cell death was influenced by the duration of melatonin exposure. Prolonged exposure to melatonin resulted in a more pronounced reduction in cell viability (Fig. 1B and D). These findings suggest that neither Raji cells nor primary T-cells respond to low melatonin concentrations, but high melatonin concentrations induce cell death. Based on these findings, we selected a maximum concentration of 1 mM melatonin for subsequent experiments, as it did not significantly affect the viability of Raji cells or T cells.

### Effects of melatonin on T-cell activation in vitro

**Fig. 2:**
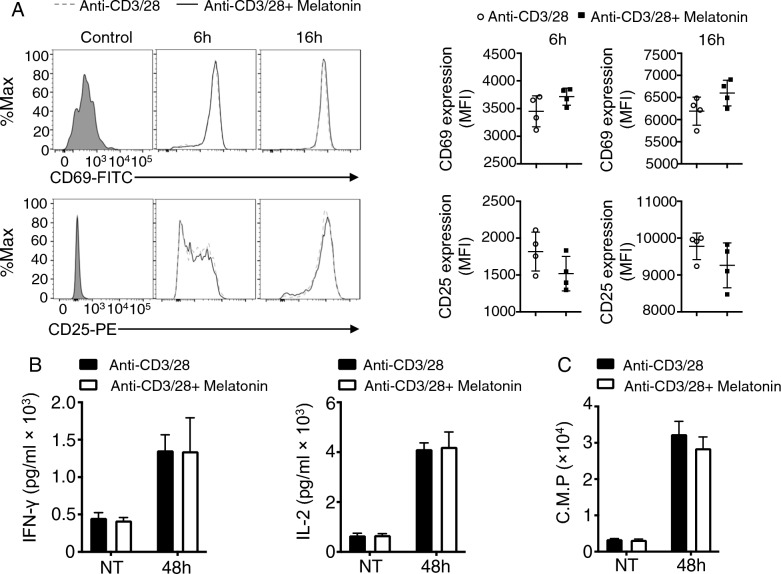
Effects of melatonin on T-cell activation in vitro. **A** Flow cytometry analysis of CD25 and CD69 expression in activated T cells treated with melatonin, as well as in control naïve T cells. **B** ELISA analysis of interferon-gamma (IFN-γ) and IL-2 production in melatonin-treated and control groups. **C** T-cell proliferation assays were conducted by pulse-labeling the stimulated T cells with [3H]thymidine for 8 h. Thymidine incorporation was measured as an indicator of cell proliferation. Data are representative of 3 independent experiments. Summary graphs (N = 4) are presented as mean ± SEM, and P values were determined by unpaired two-tailed Student’s *t*-test

T-cell activation plays a crucial role in anti-tumor immunity because activated T-cells are key effectors in tumor cell recognition and elimination [23]. To investigate the effects of melatonin on T cell activation, we treated T cells with 1 mM concentrations of melatonin and examined the impact of melatonin on T-cell activation markers and cytokine production in vitro. Flow cytometry analysis of CD25 and CD69 expression revealed no significant differences in the percentage of CD25 + and CD69 + T cells between the melatonin-treated and control groups (Fig. 2A). This suggests that melatonin does not have a noticeable effect on the expression of T-cell activation markers. Furthermore, we assessed T cell proliferation and measured the production of cytokines associated with T cell activation, including interleukin-2 (IL-2) and interferon-gamma (IFN-γ). Proliferation and ELISA assays revealed no significant differences in IL-2 and IFN-γ proliferation and secretion between the melatonin-treated and control groups (Fig. 2B and C). These findings indicated that melatonin treatment did not cause significant changes in proliferation and cytokine production associated with T cell activation. Overall, our results suggest that melatonin has no significant effect on T cell activation in vitro, as evidenced by the absence of changes in activation marker expression, proliferation, and cytokine production.

### Equivalent tumor cell cytotoxicity and CAR-T cell expansion through melatonin supplementation in vitro

**Fig. 3:**
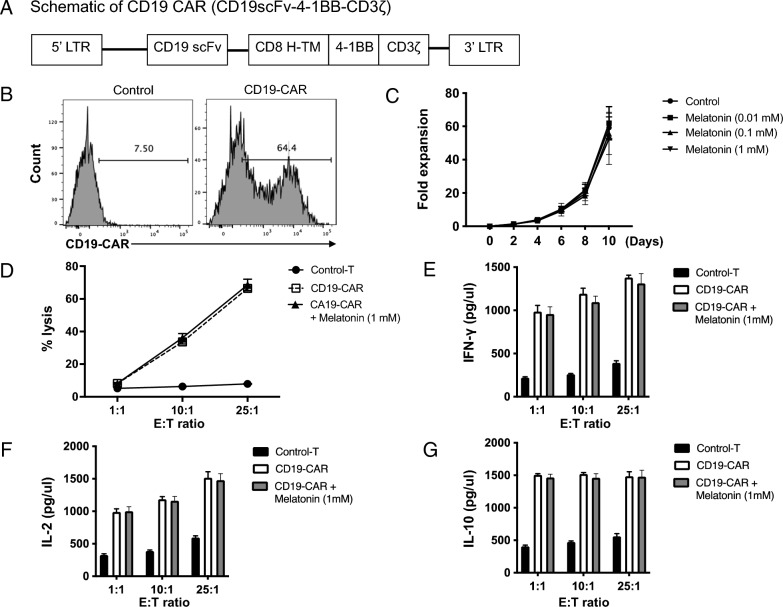
Impact of melatonin on tumor cell cytotoxicity and expansion of CD19 CAR-T cells in vitro. **A** Schematic representation of the CD19-CAR construct incorporating 4-1BB co-stimulation (CD19scFv-4-1BB-CD3ζ). **B** Flow cytometry analysis of the percentage of T cells expressing CD19 CAR on their surface. **C** Expansion potential of CAR-T cells in the presence of melatonin. **D** Cytotoxicity assay of CAR-T cells against CD19 + B cells (Raji cells) at varying effector:target (E:T) ratios. **E–G** Cytokine production analysis by CD19 CAR-T cells in the supernatant of effector-target cell co-cultures. Data are representative of 2 independent experiments. Summary graphs (N = 5) are presented as mean ± SEM

To investigate the potential impact of melatonin on tumor cell cytotoxicity and the expansion of chimeric antigen receptor CAR-T cells, we used a CD19-CAR construct incorporating 4-1BB co-stimulation (CD19scFv-4-1BB-CD3ζ, as depicted in Fig. 3A). Clinical trials have demonstrated that this CAR design achieves high rates of complete remission [24]. The CD19scFv-4-1BB -CD3ζ CAR construct was used in subsequent experiments and is hereafter referred to as CD19 CAR. Through recombinant Fc/CD19 staining, we determined that more than 60% of the T cells expressed CD19 CAR on their surface (Fig. 3B).

Our initial aim was to investigate the expansion potential of CAR-T cells in the presence of melatonin. Interestingly, the addition of melatonin to the culture medium did not augment CAR-T cell expansion (Fig. 3C). Next, we evaluated the cytotoxicity of CAR-T cells in the presence of melatonin. We co-cultured anti-CD19 CAR T cells with malignant CD19 + B cells (Raji cells) using varying effector:target (E:T) ratios for 4 h (Fig. 3D). Our findings revealed that anti-CD19 CAR-T cells exhibited specific cytotoxicity against Raji cells, indicating that they are effective at targeting CD19-expressing tumor cells. Notably, the presence of melatonin in the culture medium had no significant effect on CAR-T cell cytotoxicity. The percentage of tumor cell death by melatonin-supplemented CAR-T cells was comparable to that of CAR-T cells without melatonin supplementation (p > 0.05), indicating that the two experimental groups had comparable levels of tumor cell cytotoxicity.

Finally, after a 16-h incubation period, we examined cytokine production by CD19 CAR-T cells in the supernatant of effector-target cell co-cultures, which was subsequently analyzed using ELISA. First, we compared the levels of cytokines in the supernatants of melatonin co-cultures containing CD19 CAR or control T cells. Interestingly, the addition of melatonin did not increase IFNγ, IL-2, or IL-10 production by the treated cells (Fig. 3E–G). Taken together, these findings suggest that adding melatonin to the culture medium does not compromise the expansion ability or cytotoxicity of CAR-T cells targeting CD19-expressing tumor cells.

### Melatonin attenuates CAR T cell-induced CRS and enhances overall survival in mouse model

**Fig. 4:**
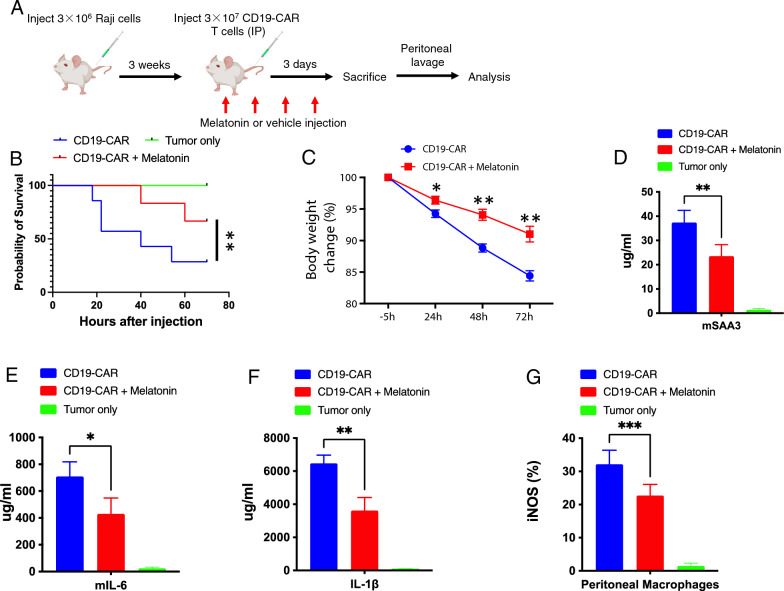
Melatonin attenuates CAR T cell-induced crs and enhances overall survival in mouse model. **A** Mouse model of CAR T cell-induced cytokine release syndrome (CRS) using SCID-beige mice. **B** Kaplan–Meier survival curve showing significantly prolonged overall survival (OS) in mice (N = 10) treated with the combination of CD19 CAR-T cells and melatonin compared to those receiving CD19 CAR-T cells alone. **C** Comparison of weight loss between melatonin-treated group and control group (N = 6). **D**–**F** Evaluation of serum cytokines on day 4 revealed a significant reduction in melatonin-treated mice (N = 6). **G** Investigation of inducible nitric oxide synthase (iNOS) activity in macrophages during CRS. Data are representative of 2 independent experiments. *P < 0.05; **P < 0.01; ***P < 0.001

Next, we aimed to mimic the clinical setting by modeling CAR T cell-induced cytokine release syndrome (CRS) in mice, in which CD19 CAR-T cells experience a high tumor burden and initiate CRS within a few days. We used SCID-beige mice to model CRS, which replicates the key aspects of CAR T cell-induced CRS observed in a clinical setting [25] (Fig. 4A). Remarkably, the combination of CD19 CAR-T cells and melatonin resulted in significantly longer overall survival (OS) compared to the mice that received CD19 CAR-T cells alone (median OS 54.7 h vs. 80.9 h, p < 0.05; Fig. 4B). Furthermore, the melatonin-treated group lost less weight (Fig. 4C). Of particular significance, the evaluation of serum cytokines on day four revealed a significant reduction in the melatonin-treated mice (Fig. 4D). Notably, CAR T cell-released cytokines such as IL-6 and IL-1β were significantly lower compared to those in the control group (Fig. 4E and F).

IL-6 and IL-1β from macrophages are key drivers of both CRS and CRES and cause CAR-T cell therapy failure and death, limiting the broad applicability of this treatment [25]. To further elucidate the role of melatonin in modulating macrophage contribution to CRS, we investigated the activity of inducible nitric oxide synthase (iNOS), an enzyme predominantly expressed by activated macrophages that is induced by IL-6 and IL-1β [26]. Consistent with previous results, we found that peritoneal macrophages significantly increased iNOS production during CRS [25]. Notably, melatonin-treated macrophages produced significantly less iNOS during CRS (Fig. 4G). These findings support the idea that the observed decrease in CRS in vivo could be attributed, at least partially, to the direct modulation of macrophage cytokine production by melatonin.

### *Equivalent antitumor efficacy and persistence of CD19 chimeric antigen receptor T cells through melatonin supplementation *in vivo

**Fig. 5:**
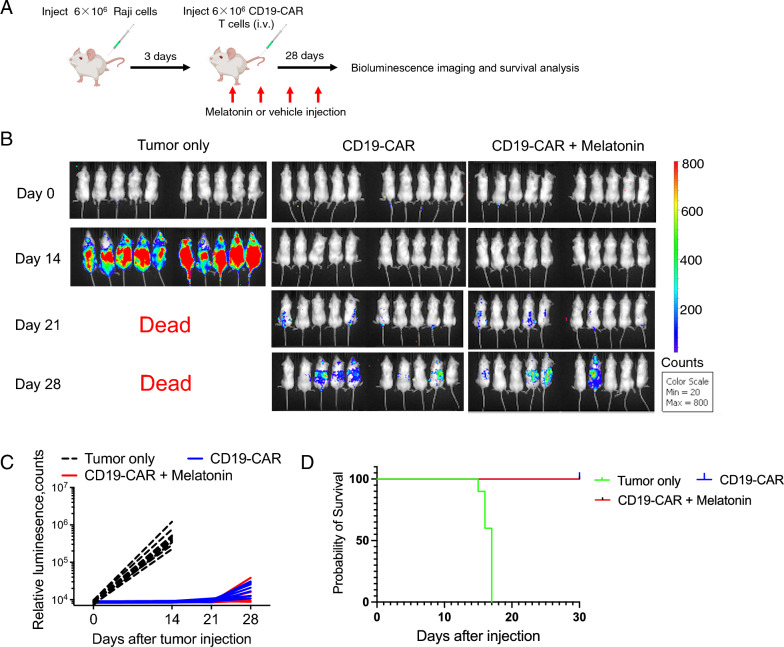
Equivalent antitumor efficacy and persistence of CD19 CAR-T cells through melatonin supplementation in vivo. **A** Experimental design illustrating the comparison between groups receiving CD19 chimeric antigen receptor (CAR) T cells with or without melatonin supplementation in a mouse model of CD19-positive tumor xenografts (N = 10). **B**, **C** Assessment of tumor growth inhibition and overall tumor burden in indicated groups (N = 10). **D** Survival rate analysis of indicated groups (N = 10). Data are representative of 2 independent experiments

Finally, we investigated the effects of melatonin supplementation on the antitumor efficacy and persistence of CD19 CAR-T cells in vivo. We used a mouse model of CD19-positive tumor xenografts and compared the outcomes between groups that received CAR-T cells with or without melatonin supplementation (Fig. 5A). Remarkably, both groups showed comparable antitumor efficacy and persistence (Fig. 5B), as well as tumor growth inhibition and overall tumor burden (Fig. 5C). Most importantly, the survival rate of the mice receiving melatonin was similar to that of the group treated solely with CAR-T therapy (Fig. 5D). These results highlight the potential of melatonin as an adjuvant therapy to improve the therapeutic outcomes of CD19 CAR T cell-based treatments, without exerting a negative impact on the antitumor efficacy or persistence of CD19 CAR-T cells.

## Discussion

Despite rapid advancements in CAR-T cell therapy, CRS management remains a significant challenge [1]. In this study, we investigated the potential of melatonin as an adjunctive therapy for managing CRS associated with CD19-targeting CAR T-cell therapy. Our findings indicate that melatonin, with its well-established immunomodulatory properties and favorable safety profile, appears to be promising in this context.

Importantly, melatonin supplementation did not compromise CD19 CAR-T cell antitumor efficacy. Tumor growth inhibition and overall tumor burden reduction were comparable between the melatonin-supplemented and control groups, indicating that melatonin does not interfere with the ability of CD19 CAR-T cells to effectively target and eliminate CD19-positive tumor cells. This finding suggests that melatonin can be safely administered as an adjuvant therapy without compromising the therapeutic potential of CAR-T cells.

**Fig. 6:**
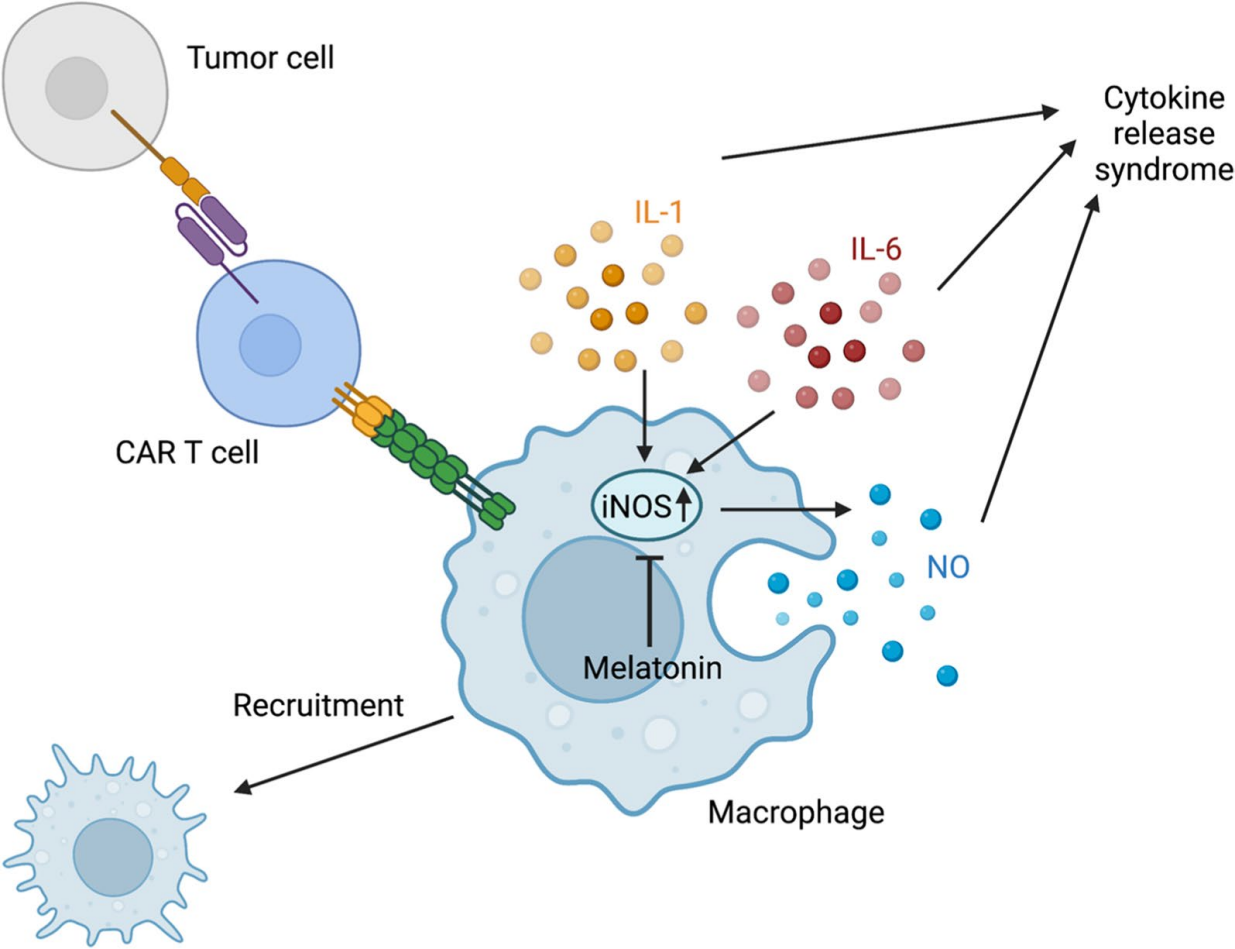
Proposed model of melatonin’s effect on cytokine release syndrome

In the SCID-beige mouse model of CRS, the combination of CD19 CAR-T cells and melatonin resulted in prolonged survival, reduced systemic toxicity, and lower levels of pro-inflammatory cytokines such as IL-6 and IL-1β. These findings highlight melatonin’s immunomodulatory effects in mitigating CRS and its potential as a therapeutic strategy. Furthermore, melatonin directly modulated macrophage activity, including the suppression of iNOS production. This provides insight into the mechanisms by which melatonin helps to reduce CRS by regulating macrophage function (Fig. 6).

There were some limitations in this study. Firstly, the specific focus of our investigation on CD19 CAR-T cells and CD19-positive tumor models may limit the generalizability of our results. The effects of melatonin observed in this study may not directly apply to CAR-T cells targeting different antigens or in the context of other tumor models. Therefore, it is crucial for future research to expand the scope and assess the effects of melatonin supplementation in diverse CAR-T cell therapies and tumor models. Furthermore, while our study suggests that melatonin has the potential to modulate macrophage function and attenuate the release of pro-inflammatory cytokines, the underlying mechanisms of these effects remain incompletely understood. Elucidating the precise signaling pathways and molecular mechanisms involved is essential for optimizing the therapeutic use of melatonin in CRS in the future. Additionally, as we explore the potential of melatonin, it is important to recognize that various interventions, including cytokine blockade, corticosteroids, and other immunomodulators, have been employed in clinical practice. Therefore, future studies should aim to directly compare melatonin with other treatments to determine its relative efficacy, safety, and appropriateness in different clinical scenarios, including its potential as a preventive option. Lastly, an important consideration in our study is the absence of a concurrent model of CRS and disease response in CAR-T cell therapy. Future research should aim to bridge this gap by developing preclinical models that replicate CRS and disease response simultaneously. Such models would allow for a more comprehensive assessment of the impact of immunomodulators like melatonin on both the safety and therapeutic efficacy of CAR-T cell therapies.

In conclusion, our study demonstrates that melatonin supplementation does not compromise the antitumor efficacy or persistence of CD19 CAR-T cells in a mouse model of CD19-positive tumors. Moreover, melatonin supplementation can reduce CRS-associated toxicity and prolong overall survival. While our study provides important foundational insights, the applicability of our findings to human CAR-T cell therapy and CRS management requires further investigation. Clinical trials specifically designed to evaluate melatonin's efficacy and safety in human subjects, considering appropriate dosing and administration schedules, are essential for establishing its clinical relevance.

## Data Availability

The datasets used and/or analyzed during the current study are available from the corresponding author on reasonable request.
